# Supplementary material for: Heliorhodopsin-mediated light-modulation of ABC transporter
Source: Nat Commun. 2024 May 21;15:4306. doi: 10.1038/s41467-024-48650-1 (PMC11109279; doi:10.1038/s41467-024-48650-1)
Supplement: Supplementary file 1 — Supplementary Information [file 41467_2024_48650_MOESM1_ESM.pdf]

Supplementary Information

Heliorhodopsin-mediated light-modulation of ABC transporter

Shin-Gyu Cho,[1,2]† Ji-Hyun Kim,[1]† Ji-eun Lee,[1] In-Jung Choi,[1] Myungchul Song,[1] Kimleng Chuon,[1] Jin-gon Shim,[1] Kun-Wook Kang,[1] and Kwang-Hwan Jung[1]*

[1] Department of Life Science, Sogang University, Seoul, Korea,

[2] Research Institute for Basic Science, Sogang University, Seoul, Korea

* Corresponding author, E-mail address: kjung@sogang.ac.kr

† These authors contributed equally to this work.

This file includes:

Supplementary Discussion

Supplementary Methods

Supplementary Figs. 1–14

Supplementary Tables 1–3

References (1–12)

Supplementary Discussion

Predicted crystal structure of OmrDE

To investigate protein–protein interactions between purified OmrDE and OcHeR using ITC analysis, we considered various conformations of ABCTs. ABCTs undergo conformational changes, transitioning from inward-facing (IF) to outward-facing (OF) conformations through ATP binding and hydrolysis[1]. The IF conformation comprises two distinct forms: the narrow separation of NBDs under apo condition (IF_{narrow}) and the wide separation of NBDs (IF_{wide}). Notably, substrate binding is specifically associated with the IF_{wide} conformation[2]. It is conceivable that the IF_{narrow} conformation in purified protein may more frequent than the IF_{wide} conformation in the absence of substrate due to release and dissociation. Consequently, when the purified protein is in a substrate-deficient solution, it may predominantly adopt the IF_{narrow} conformation. Therefore, we assumed that the conformation of purified OmrDE is the IF_{narrow} conformation rather than the IF_{wide} conformation.

The protein sequences of OmrD and OmrE were analyzed using Swiss-Model[3,4]. Subsequently, we chose the available protein crystal structure TmrAB inward-facing conformation (PDB code: 6RAF) for heterodimeric OmrDE, which considered the identity, Global Model Quality Estimate (GMQE), and Quaternary Structure Quality Estimate (QSQE) scores[2–4]. The identity, GMQE, and QSQE scores of OcHeR to HeR-48C12 were 62.06, 0.84, and 0.74, respectively. The identity, GMQE, and QSQE scores of OmrDE to TmrAB inward-facing conformation were 30.21, 0.67, and 0.61, respectively. The relatively low identity score of OmrDE may be owing to the diversity of amino acid sequence in the TMDs of ABCTs. Thus, considering the three scores and the IF_{narrow} conformation, structural analysis using the crystal structure TmrAB template was considered.

Enthalpic and entropic influences

The enthalpic factor contributes favorably to specific non-covalent interactions (hydrogen bond or polar interaction), whereas the entropic factor favors interactions that tend to be more hydrophobic[5]. The ΔH and $-T\Delta S$ values of OcHeR WT for OmrDE were positive and negative values, respectively (Fig. 6a), indicating unfavorable non-covalent interactions and favorable hydrophobic interactions. The favorable hydrophobic interactions may be attributed to the binding between membrane proteins. However, the absence of positively charged residues in ICL1 suggests that certain residues may interfere with polar interactions, leading to non-binding results. The ΔH and $-T\Delta S$ values of mutants with decreased K_d values—W225A, R229Q, and R235Q—were lower and higher than that of WT, respectively, indicating more favorable non-covalent interactions than that of WT (Figs. 4a,6a). Additionally, the ΔG values of the mutants were similar to WT (Fig. 6a), suggesting a ubiquitous phenomenon of enthalpy–entropy compensation by enthalpy gains and entropy losses. Therefore, the mutants formed new hydrogen bonds or other polar interactions, decreasing conformational entropy.

The comparison between the two results—1) the unfavorable non-covalent interaction and the favorable hydrophobic interaction, and 2) the influence of non-covalent interaction via positively charged residues in ICL1—reveals inconsistency. The presence of both attractant

residues (R102, R104, R105, D167, and W170) and repellent residues (W225, R229, R233, and R235) in OcHeR may account for the two results. Consequently, the charge-charge interplay potentially leads to compensatory effects, thus presenting an unclear discernment. Although the compensatory effects appeared, the reason for the binding may be that the attractant residues are stronger than the repellent residues. In particular, the R233Q for OmrDE resulted in sequential binding sites as positive cooperativity where the first molecule binds to the first or empty site and the second molecule has enhancing binding affinity to the second site. The K_d value of R233Q-2 (second binding) was 1.7-fold lower than that of R233Q-1 (first binding) (Fig. 4a). The ΔH and $-T\Delta S$ values of R233Q-1 were similar to those of W225A, R229Q, and R235Q mutants, while those of R233Q-2 were similar to those of WT (Fig. 6a). R233Q faces the outside, not the interface between membrane proteins, implying that the repellent effect is smaller than W225, R229, and R235 (Fig. 4d). Therefore, the absence of positively charged residue in R233 reveals the sequential binding.

Consequently, the binding affinities of the OcHeR mutants were influenced by the charged and aromatic residues of ICLs, and the structure of the homodimeric OcHeR may affect the binding affinity of OmrDE. Moreover, the positively charged residues in ICL1 may allow the reduction of the ubiquitous phenomenon of enthalpy–entropy compensation by decreasing the high ΔH value of proteins, resulting in the non-spontaneous reaction becoming spontaneous, as a positive ΔG value changes to a negative ΔG value.

Fine-tuning OmrDE activity via OcHeR

In nature, living organisms maintain a cellular metabolism through fine-tuning regulation in a network of regulatory pathways[6,7]. The catalytic efficiencies of carbonic anhydrase with poly(γ-glutamic acid) and phosphorylated human choline kinase beta with choline were increased by 1.2 and 2.36 times, respectively[8,9]. These enhancements are crucial because many biochemical reactions in living organisms naturally occur at slow rates or require high activation energy. In our findings, we observed a significant enhancement in the activity of OmrDE binding to OcHeR, resulting in a 1.8–2.8 fold increase. This phenomenon is not uncommon in nature; therefore, the strategy of increasing OmrDE activity with OcHeR and light may be a more efficient approach to reducing the activation energy from the bacterium's perspective. In addition, fine-tuning prevents rapid ATP consumption, and enhanced OmrDE activity may be sufficient for drug resistance in the habitat. Hence, this would be preferable to solely increasing OmrDE expression to transport drugs by consuming energy.

Supplementary Methods

Plasmid preparation

The genome of the *O. cerasi* strain USBA17B2 was deposited in the National Center for Biotechnology Information (NCBI, accession number: OBQK01000028) and submitted to the Joint Genome Institute (project ID: 1094766). Genes encoding OcHeR (accession number: SOC58301) and OmrE (accession number: SOC58303) containing *Nde*I and *Not*I restriction sites with hexahistidine tags at the N-terminus of OcHeR and the C-terminus of OmrE as well as OmrD (accession number: SOC58305)-encoding genes containing *Xba*I and *Nde*I restriction enzyme sites with a ribosome binding site at the N-terminus and hexahistidine tags at the C-terminus were codon-optimized for protein expression in *E. coli* and chemically synthesized (Integrated DNA Technologies, USA). The oligomers used for preparation, and the plasmids constructed in this study are listed in Supplementary Tables 2 and 3.

The synthesized OcHeR and OmrE were cloned into the *Nde*I and *Not*I sites of the pET21b vector and named pET21b-OcHeR and pET21b-OmrE, respectively. Each point mutation in OcHeR was generated via site-directed mutagenesis, and the mutated gene sequences were cloned into the pET21b vector. The synthesized OmrD was cloned into the *Xba*I and *Nde*I sites of the pET21b vector.

To co-express OmrD, OmrE, and OcHeR in one vector, we designed the expression systems of OmrDE and OcHeR to be regulated by P_{T7} and P_{lac}, as well as P_{araBAD}, respectively. In the pET vector, the synthesized OmrD was cloned into the *Xba*I and *Nde*I sites of pET21b-OmrE, termed pET21b-OmrDE. *paraBAD* and *rrnB T1* terminator fragments were amplified via polymerase chain reaction (PCR) using the F1+R1 and F2+R2 primers in pKA001-AbGS_AbHeR [10], respectively, and the fragments were amplified using overlap extension PCR. OcHeR fragments were amplified via PCR using the F3+R3 primers. Lastly, the amplified fragment was introduced into the *Bgl*II site of pET21b-OmrDE, whereas the OcHeR fragment was cloned into the *Bmt*I and *Pme*I sites of pET21b-OmrDE, which was termed pET21b-DEH.

OmrE D577Q was prepared using site-directed mutagenesis, and the mutated gene sequences were cloned into pET21b-DE, termed pET21b-DE^{m}. To truncate the NBD of ABCT, a fragment was amplified via PCR using the F4+R4 primers in pET21b-OmrE and introduced into the *Nde*I and *Xho*I sites of pET21b-DE. In addition, a fragment amplified via PCR using the F5+R5 primers in pET21b-OmrD was cloned into the *Eco*RI and *Nde*I sites of the introduced vector, termed pET21b-$D^{tc}E^{tc}$.

To perform the His-tag pull-down assay, pET21b-OmrE was amplified via PCR using the F6+R6 primers. Subsequently, the PCR product was digested with *Sph*I and *Xho*I restriction enzymes and cloned into the *Sph*I and *Xho*I sites of pACHA-mScarlet encoding the p15A origin, P_{T7}::*lacO*::RBS::3xHA-tag::mScarlet::T7 terminator, *lacI*, and *CmR*, which was termed pACHA-OmrE.

In the pKA vector, a fragment digested with *Not*I and *Xba*I restriction enzymes from pET21b-DE was introduced into the *Not*I and *Xba*I sites of the pKA001-AbGS_AbHeR vector. Subsequently, a fragment digested with *Pme*I and *Age*I restriction enzymes from pET21b-DEH was cloned into the *Pme*I and *Age*I sites of the introduced vector, which was termed pKA001-DEH. A fragment digested with *Xba*I and *Not*I restriction enzymes from pKA001-DE^{m} was

cloned into the *Xba*I and *Not*I sites of pKA001-DEH, termed pKA001-DE^{m}H. pKA001-DEH^{m} and pKA001-DE^{m}H^{m} were generated via site-directed mutagenesis.

Protein expression and purification

Membrane protein expression and purification procedures were performed as previously described[11]. OcHeR WT and mutants were expressed in *E. coli* C43 (DE3) harboring pET21b-H and pET21b-H$^{R102Q,\ R104Q,\ R105Q,\ D167N,\ W170A,\ W225A,\ W229A,\ R233Q,\ or\ R235Q}$ in LB medium containing 50 μg/mL ampicillin, 0.84 mM isopropyl β-D-1-thiogalactopyranoside (IPTG) for induction, and 7 μM all-*trans*-retinal (ATR, Toronto Research Chemicals, Canada) at 37 °C and 200 rpm. To express Omr transporters (OmrD, OmrE, OmrDE, and OmrD^{tc}E^{tc}), we cultured *E. coli* C43 (DE3) harboring plasmids (pET21b-DE, pET21b-D, pET21b-E, and pET21b-D^{tc}E^{tc}) at 37 °C and 200 rpm in LB medium containing 50 μg/mL ampicillin, followed by induction with 0.84 mM IPTG with and without 7 μM ATR and 0.2% (w/w) L-arabinose. Subsequently, the cells were incubated at 18 °C and 240 rpm for 24 h. Induced cells were harvested and washed with buffer S (150 mM NaCl and 50 mM Tris–HCl, pH 7.0).

Cells expressing OcHeR and Omr transporters were resuspended in buffer S containing 1 mM phenylmethylsulphonyl fluoride (PMSF) as well as 0.5 mM PMSF, 1 μM pepstatin A, and 10 μM leupeptin, respectively, and subsequently disrupted by sonication. Non-disrupted cells and debris were removed by centrifugation, and membrane fractions were isolated by ultracentrifugation at 100,000 ×*g* and 4 °C for 1 h (L-90 ultracentrifuge, Beckman, USA). Membrane fractions were solubilized in buffer S containing 1% (w/w) *n*-dodecyl-β-D-maltopyranoside (DDM, Goldbio, USA). The solubilized membrane proteins were centrifugated at 30,000 ×*g* and 4 °C for 30 min. The supernatants were subjected to affinity chromatography using Ni^{2+}-NTA agarose (Qiagen, USA). The membrane proteins were washed and eluted by adding buffer SD (150 mM NaCl, 50 mM Tris–HCl at pH 7.0, and 0.02% (w/w) DDM) containing 25 mM imidazole and 250 mM imidazole, respectively. The buffer of the eluted OcHeR and Omr transporters was exchanged with buffer SD using Amicon Ultra-4 10,000 and 50,000 MWCO centrifugal filter units, respectively. The Amicon Ultra-4 10,000 MWCO centrifugal filter unit was used for OmrD^{tc}E^{tc}.

Sodium dodecyl sulphate polyacrylamide gel electrophoresis (SDS–PAGE) and western blot analysis

Membrane proteins quantified using the Bradford assay were added to Laemmli sample buffer and incubated at 25 °C for 30 min. The denatured samples were subjected to discontinuous SDS-PAGE, where the acrylamide concentrations of the stacking and separating Tris-Glycine gels were 5% and 12 or 15%, respectively. After electrophoresis, the gels were immersed in a staining solution (0.05% (v/v) Coomassie brilliant blue R-250, 50% (v/v) MeOH, and 10% (v/v) glacial acetic acid) for gel staining and subsequently incubated for 2 h. The stained gels were then washed with a destaining solution (40% (v/v) MeOH and 10% (v/v) glacial acetic acid).

To develop western blot, the gels after electrophoresis were transferred to polyvinylidene

difluoride (PVDF) membrane layered with filter paper wetted with transfer buffer (20% (v/v) MeOH, 25 mM Tris-base, 192 mM glycine, 0.1% SDS, and pH 8.3). The proteins on the gel were transferred onto the PVDF membrane using semi-dry transfer devices (ATTO, Japan) with 144 mAh for 20 to 30 min. The transferred PVDF membranes were then incubated in TBS-T buffer (20 mM Tris-HCl at pH 7.6, 137 mM NaCl, 0.15% (v/v) Tween-20) containing 5% (w/w) skim milk and subsequently rocked for 1 h at room temperature. After removing the buffer, the PVDF membranes were added to TBS-T buffer containing 5% (w/w) skim milk.

Primary antibodies [rabbit anti-hexahistidine (His)-tag (GTX115045, GenTex, USA) and mouse anti-trihemagglutinin (HA)-tag (SC-7392, Santa Cruze, USA)] were treated to PVDF membranes in TBS-T buffer containing 5% (w/w) skim milk at a ratio of 1:10,000. After treatment, the PVDF membranes were rocked for 16 h at 4 °C. After removing the buffer, the PVDF membranes were washed three times with TBS-T buffer. Secondary antibodies [goat HRP-conjugated anti-rabbit antibody for His-tag (GTX213110-01, GenTex, USA) and goat HRP-conjugated anti-mouse antibody for HA-tag (SC-2005, Santa Cruze, USA)] were treated to the PVDF membranes in TBS-T buffer containing 5% (w/w) skim milk at a ratio of 1:7,500. After treatment, the PVDF membranes were rocked for 45 min at room temperature. The PVDF membranes were then washed three times with TBS-T buffer. The PVDF membranes were dropped to ECL substrate solution and subsequently exposed to X-Ray film using developer (X-Ray film processor Pro CQ, Daesung Tech, Japan).

Searching for bacterium habitat

O. cerasi strain USBA17B2 (Joint Genome Institute (JGI) Project Id: 1094766) was isolated in a salt mine in Colombian Andes Mountain, which was reported in a study titled "Comparative analysis of microorganisms from saline springs in Andes Mountain Range, Colombia" (Integrated Microbial Genomes and Microbiomes in JGI, Award DOI: 10.46936/10.25585/60000546); however, the study has not been published. Therefore, we searched for research articles containing microorganisms deposited in the study to obtain the correct isolation environment. The microorganisms *Isoptericola halotolerans* CG 23 (JGI Project Id: 1094744), *Nesterenkonia sandarakina* CG 35 (JGI Project Id: 1094746), and *Oceanibaculum indicum* USBA 36 (JGI Project Id: 1094782) were reported[12]. The isolation environment was the Zipaquirá salt mine in Colombia. Cytotoxic compounds in the habitat were identified via liquid chromatography–mass spectrometric analysis[12] after performing cytotoxic activity assays using human cancer cell lines.

Supplementary Figures and Tables

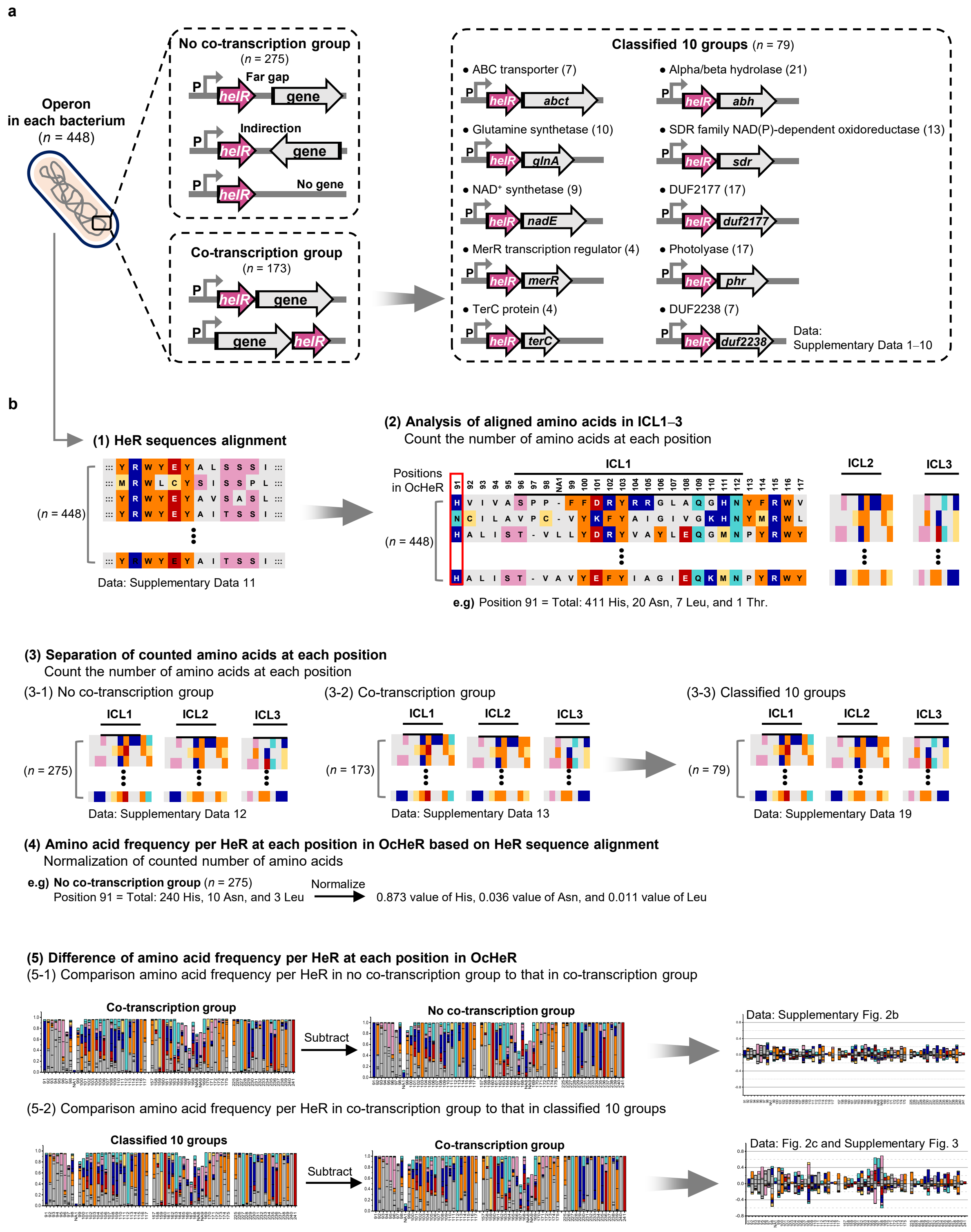

Supplementary Fig. 1. Schematic procedures of analyzing genes in operon and amino acid frequency.

Each figure or data of the analysis is labeled by each step in the procedures. **a** Analyzing *helR* and neighboring gene in the operon of each bacterium. The operons were classified into three

groups: 1) Non-co-transcription group, two or more genes cannot be continuously transcribed by RNA polymerase at a single promoter. 2) Co-transcription group, two or more genes can be continuously transcribed by RNA polymerase at a single promoter. 3) In classified 10 groups, frequent neighboring genes flanking *helR* are classified groups of the neighboring gene-containing operon. The total number of the operon in each group are indicated by parenthesis near protein labels. **b** Analyzing procedures of amino acid frequency per HeR at each position in OcHeR. (1) HeR sequences deposited in NCBI are aligned using MUSCLE. (2) Amino acids at each position in ICL1–3 of OcHeR are counted and summed (e.g. a total of 411 His, 20 Asn, 7 Leu, and 1 Thr counts at position 91). (3) A total of amino acids at each position are counted by HeR sequence alignments of the non-co-transcription group (3-1), co-transcription group (3-2), and classified 10 groups (3-3). (4) In each group, the total amino acids are normalized by the number of HeR sequences in each group, labeling amino acid frequency per HeR. (e.g. 0.873 value of His means that 87.3% of HeRs in the non- co-transcription group contains His at position 91). (5) Each amino acid frequency per HeR in those groups are subtracted using the two compared groups.

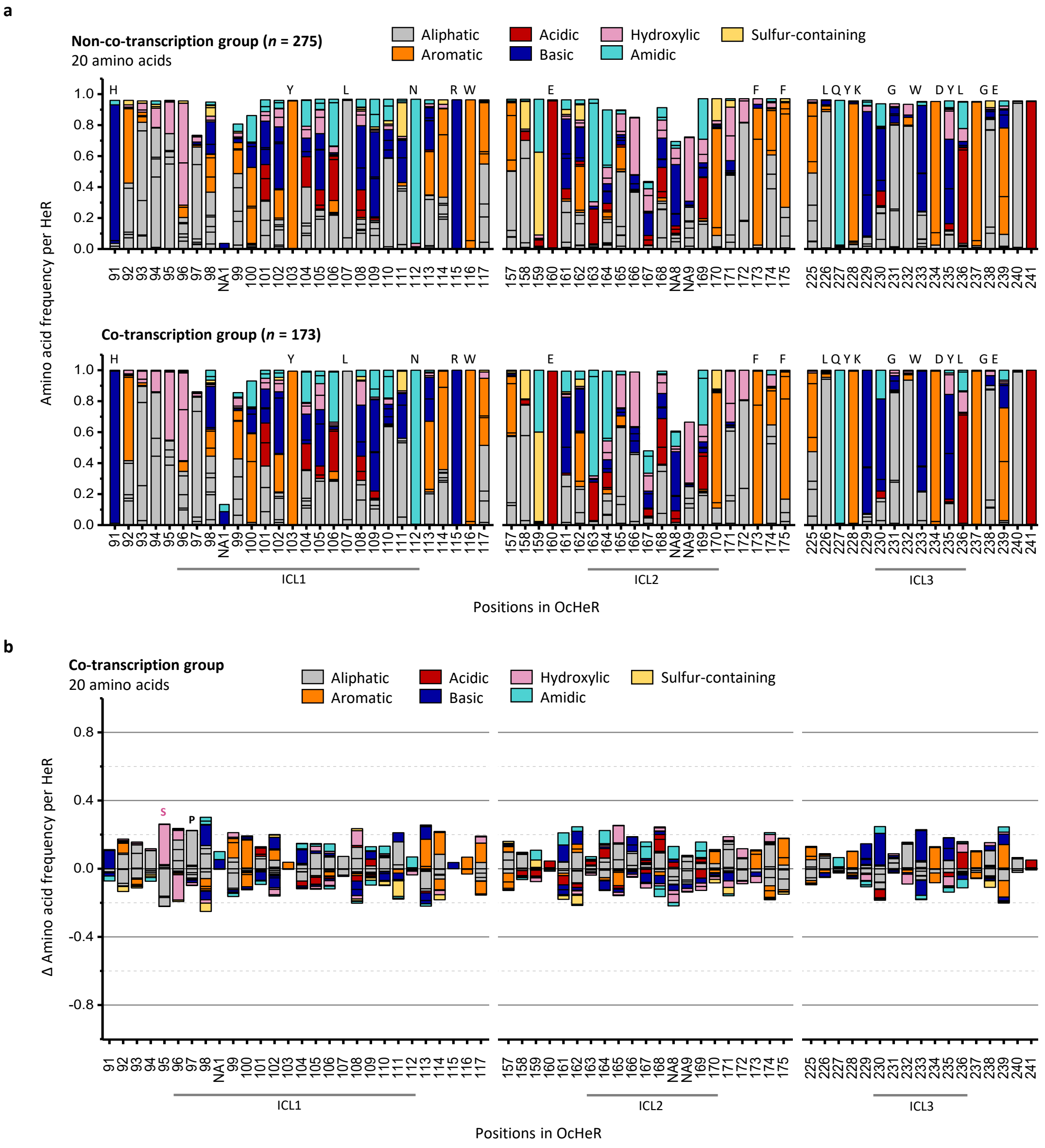

Supplementary Fig. 2. Analyzing amino acid frequency per HeR at each position in OcHeR based on HeR sequence alignment.

a The amino acids of each HeR at the residue positions in OcHeR are counted and subsequently normalized by the number of HeRs; that is, amino acid frequency per HeR. Highly conservative residues are marked on stack-bars. The non-aligned positions in the amino acid alignment data are indicated using NA. **b** The difference of 20 amino acids sequences of HeRs in a co-transcription group (*helR* and neighboring genes) compared to a no co-transcription group (*helR* alone). All data are analyzed based on the steps shown in Supplementary Fig. 1b and data in Supplementary Data 11–13.

		Aliphatic						Aromatic			Acidic		Basic			Hydroxylic		Sulfur-contaiing		Amidic	
		A	G	I	L	P	V	F	W	Y	D	E	R	H	K	S	T	C	M	N	Q
Helix 2	91	0.000	-0.018	0.000	-0.018	0.000	0.000	0.000	0.000	0.000	0.000	0.000	-0.018	0.124	0.000	0.000	0.000	0.000	0.000	-0.029	0.000
	92	0.117	-0.009	-0.002	-0.195	0.000	0.018	0.166	-0.007	0.000	0.000	0.000	0.000	-0.004	0.000	0.000	-0.015	-0.029	0.000	0.000	0.000
	93	-0.058	-0.007	-0.045	0.106	0.000	0.097	-0.011	-0.007	-0.009	0.000	0.000	0.000	0.000	0.000	-0.015	0.007	0.000	-0.015	0.000	0.000
	94	-0.011	0.000	-0.189	0.099	-0.011	0.140	0.000	0.000	-0.004	0.000	0.000	0.000	0.000	0.000	0.000	0.066	-0.011	-0.015	-0.022	0.000
	95	-0.309	0.108	0.027	-0.007	0.000	-0.046	0.000	0.000	0.000	0.000	0.000	0.000	0.000	0.000	0.277	-0.007	-0.004	0.000	0.000	0.000
ICL1	96	0.076	-0.025	0.155	0.023	-0.007	0.056	-0.015	0.000	-0.015	0.000	0.000	0.000	0.000	0.000	0.018	-0.221	0.000	-0.002	0.000	0.000
	97	-0.016	0.009	0.002	0.029	0.305	0.014	-0.011	0.000	0.000	0.000	0.000	-0.007	0.000	-0.015	-0.033	-0.007	0.000	0.000	-0.004	0.000
	98	-0.020	0.177	-0.036	-0.062	0.040	-0.136	-0.098	-0.022	-0.022	0.000	-0.004	-0.062	0.000	0.158	-0.011	-0.025	-0.055	-0.015	0.009	0.045
	NA1	0.000	0.000	0.000	0.000	0.000	0.000	0.000	0.000	0.000	0.000	0.000	-0.004	0.000	0.056	0.000	0.000	0.000	0.000	0.089	0.000
	99	-0.025	-0.140	0.086	0.214	0.000	-0.028	0.088	-0.007	-0.040	0.000	0.000	-0.025	0.000	-0.007	0.009	-0.013	0.000	-0.011	-0.047	0.000
	100	-0.011	-0.004	-0.011	-0.004	0.000	-0.007	0.239	0.002	-0.200	0.000	0.000	0.030	-0.009	0.018	0.000	0.038	0.000	0.000	0.057	0.000
	101	0.052	-0.073	0.000	0.000	-0.022	0.000	0.000	0.000	-0.007	0.001	0.099	-0.022	-0.011	0.019	-0.024	0.050	0.000	0.000	0.005	-0.031
	102	-0.002	0.027	0.092	0.003	-0.011	-0.007	-0.011	0.033	-0.004	0.000	-0.004	0.000	-0.011	-0.098	0.077	-0.042	0.000	-0.004	0.014	-0.015
	103	0.000	0.000	0.000	0.000	0.000	0.000	0.000	0.000	0.044	0.000	0.000	0.000	-0.004	0.000	0.000	0.000	0.000	0.000	0.000	0.000
	104	-0.049	-0.015	-0.009	-0.018	0.000	-0.131	0.000	0.000	0.000	0.013	-0.019	0.018	0.000	0.153	-0.004	0.003	0.000	0.000	0.099	-0.004
	105	0.174	-0.006	-0.040	0.000	0.000	0.000	0.000	0.000	-0.007	0.013	-0.086	0.008	0.009	-0.077	0.008	0.092	0.000	-0.004	-0.040	-0.013
	106	-0.004	0.039	0.000	-0.004	-0.004	-0.007	0.000	-0.004	-0.064	0.013	-0.022	-0.004	-0.007	-0.011	-0.027	0.051	-0.004	0.000	0.112	-0.022
	107	-0.004	0.000	-0.069	0.113	0.000	0.000	0.000	0.000	0.000	0.000	0.000	-0.007	0.000	0.000	0.000	0.000	0.000	0.000	0.000	0.000
	108	0.139	-0.018	-0.007	0.031	0.000	0.043	-0.011	0.000	0.000	-0.035	-0.055	-0.091	0.000	-0.197	0.260	-0.025	-0.011	0.052	-0.004	-0.038
	109	0.014	-0.031	0.000	-0.040	-0.004	-0.011	0.000	0.000	0.000	-0.015	0.018	-0.019	-0.011	0.165	0.002	0.018	0.000	-0.015	-0.017	-0.022
	110	-0.015	0.189	0.000	-0.004	0.000	0.000	0.000	0.000	0.000	0.000	0.000	-0.038	0.014	-0.056	-0.013	-0.011	-0.022	0.000	0.007	-0.018
	111	-0.016	0.000	0.262	-0.015	0.000	0.020	-0.022	0.000	0.000	0.000	0.000	-0.021	-0.024	-0.004	-0.007	-0.015	0.000	-0.117	-0.011	0.002
	112	0.000	-0.004	0.000	0.000	-0.004	0.000	0.000	0.000	0.000	-0.004	0.000	-0.004	0.000	0.000	0.000	-0.022	0.000	0.000	0.069	0.000
Helix 3	113	-0.004	-0.004	0.002	-0.004	-0.058	-0.013	0.034	0.000	0.087	-0.011	0.000	-0.088	-0.018	0.124	0.005	0.007	0.000	0.000	0.000	-0.025
	114	0.060	-0.015	-0.006	-0.058	-0.011	-0.027	0.229	0.000	-0.091	0.000	0.000	-0.004	0.000	0.000	0.000	-0.013	0.000	-0.029	0.000	0.000
	115	0.000	0.000	0.000	0.000	0.000	0.000	0.000	0.000	0.000	0.000	0.000	0.036	0.000	0.000	0.000	0.000	0.000	0.000	0.000	0.000
	116	0.000	0.000	0.000	-0.004	0.000	0.000	-0.013	0.053	0.000	0.000	0.000	0.000	0.000	0.000	0.000	0.000	0.000	0.000	0.000	0.000
	117	-0.031	0.000	-0.138	0.043	0.000	0.071	0.137	-0.015	-0.013	0.000	0.000	0.000	-0.007	0.000	0.000	-0.004	0.000	-0.007	0.000	0.000
		A	G	I	L	P	V	F	W	Y	D	E	R	H	K	S	T	C	M	N	Q
Helix 4	157	-0.026	-0.004	-0.015	0.036	0.000	-0.004	-0.031	0.130	-0.046	0.000	0.000	0.000	-0.004	0.000	-0.007	0.000	0.000	0.000	0.002	0.000
	158	-0.011	0.000	-0.016	-0.001	0.000	0.086	0.000	0.000	0.000	-0.046	0.000	0.000	0.000	0.000	-0.004	-0.011	0.000	0.050	-0.004	-0.011
	159	-0.004	0.000	0.000	0.000	-0.004	-0.004	-0.007	0.000	0.000	0.000	-0.040	0.000	-0.007	-0.004	-0.022	0.000	0.000	0.010	0.000	0.114
	160	-0.007	0.000	0.000	0.000	0.000	0.000	0.000	0.000	0.000	0.000	0.051	0.000	0.000	0.000	0.000	0.000	0.000	0.000	0.000	-0.007
	161	-0.011	0.000	-0.033	0.048	0.000	-0.035	0.000	0.000	-0.015	0.000	-0.053	0.006	-0.029	0.078	-0.006	0.002	0.000	-0.033	0.000	0.115
	162	-0.009	-0.004	-0.062	0.148	0.000	-0.018	0.112	0.000	-0.005	-0.011	-0.022	-0.022	0.057	-0.004	-0.013	-0.018	0.000	-0.073	-0.007	-0.013
ICL2	163	0.018	-0.007	0.000	0.000	-0.011	-0.007	0.000	0.000	0.000	0.013	-0.023	0.000	0.020	0.000	0.000	-0.016	0.000	0.000	0.060	-0.013
	164	0.050	-0.004	0.005	-0.004	-0.055	0.079	-0.015	0.000	0.018	-0.011	0.045	-0.051	-0.004	-0.035	-0.036	-0.031	0.000	0.000	-0.015	0.163
	165	-0.006	0.121	-0.004	-0.029	0.090	-0.004	-0.015	0.000	-0.053	-0.015	0.000	-0.029	-0.006	-0.018	-0.022	0.102	0.000	0.000	-0.009	-0.004
	166	0.022	-0.110	0.000	0.000	0.043	0.000	0.000	0.000	0.000	0.186	0.069	-0.024	0.000	0.009	0.148	-0.355	0.000	0.000	-0.004	0.165
	167	-0.004	0.007	0.000	0.000	-0.004	0.000	0.000	0.000	0.000	0.043	-0.004	0.019	-0.004	0.309	-0.039	0.020	0.000	0.000	0.226	-0.007
	168	-0.060	0.097	0.177	-0.004	0.090	0.036	-0.022	0.000	0.000	-0.040	-0.156	-0.051	-0.004	-0.135	-0.051	0.289	0.000	0.000	-0.018	-0.062
	169	0.000	0.081	-0.018	0.000	-0.004	0.000	0.000	-0.015	0.000	-0.025	0.072	-0.058	-0.004	-0.016	-0.042	-0.048	0.000	0.000	0.004	0.101
	170	-0.004	-0.007	-0.004	0.092	0.000	0.000	-0.051	0.094	0.000	-0.004	0.000	-0.007	-0.011	-0.007	-0.022	0.000	0.000	-0.041	0.000	0.000
Helix 5	171	-0.004	0.137	-0.035	0.070	0.000	0.031	0.000	-0.007	-0.004	0.000	0.000	-0.011	0.000	-0.040	-0.046	-0.008	0.000	-0.044	0.000	-0.011
	172	0.050	0.000	0.000	-0.007	-0.085	0.013	0.000	0.000	0.000	0.000	0.000	0.000	-0.004	0.000	0.077	-0.015	0.000	0.000	0.000	0.000
	173	-0.007	0.000	0.000	-0.018	0.000	0.000	0.038	0.000	0.078	0.000	0.000	0.000	-0.025	0.000	0.000	-0.036	0.000	0.000	0.000	0.000
	174	0.000	-0.004	0.070	-0.044	0.000	-0.060	-0.029	-0.018	-0.011	0.000	0.000	-0.004	0.000	0.000	-0.004	0.157	-0.004	-0.004	-0.018	0.000
	175	-0.007	0.000	-0.064	-0.082	0.000	-0.027	0.093	0.144	-0.002	0.000	0.000	0.000	0.000	0.000	-0.004	0.000	-0.004	-0.018	0.000	0.000
		A	G	I	L	P	V	F	W	Y	D	E	R	H	K	S	T	C	M	N	Q
Helix 6	225	-0.035	-0.004	0.072	-0.089	0.000	-0.068	-0.035	0.199	0.018	0.000	0.000	-0.004	-0.004	0.000	0.000	-0.015	0.000	0.000	0.000	0.000
	226	-0.004	-0.004	0.000	0.095	0.000	0.025	-0.015	0.000	-0.022	0.000	0.000	-0.007	0.000	-0.029	0.000	-0.004	0.000	0.000	0.000	0.000
	227	0.000	-0.011	-0.004	0.000	-0.004	-0.004	0.000	0.000	0.000	0.000	-0.004	0.000	0.000	0.000	0.000	0.000	0.000	0.000	0.000	0.065
	228	-0.004	0.000	0.000	-0.025	0.000	-0.004	-0.011	-0.007	0.113	0.000	0.000	-0.011	-0.004	0.000	0.000	-0.004	0.000	0.000	0.000	0.000
	229	0.007	-0.015	0.000	-0.044	0.000	0.000	-0.007	0.000	0.000	0.000	0.000	0.064	0.000	0.107	0.000	-0.036	0.000	-0.004	-0.022	-0.011
ICL3	230	-0.011	-0.066	-0.004	-0.036	0.031	-0.004	0.000	0.000	0.000	0.000	-0.069	0.083	0.000	0.235	-0.004	0.000	0.000	-0.004	-0.007	-0.082
	231	-0.011	-0.004	-0.018	-0.011	0.000	0.066	0.009	0.002	0.000	0.000	0.000	0.003	-0.011	-0.002	0.056	-0.018	0.000	0.000	0.000	0.000
	232	0.000	0.195	0.000	0.000	0.000	-0.004	-0.004	-0.038	0.000	0.000	0.000	-0.015	0.000	0.000	-0.069	0.000	0.000	0.000	0.000	0.000
	233	-0.015	-0.022	-0.025	-0.004	0.050	-0.022	-0.004	0.000	0.000	0.000	0.000	-0.117	-0.004	0.235	0.000	0.000	0.000	0.000	-0.022	-0.004
	234	-0.004	0.000	0.000	0.000	-0.022	0.000	-0.042	0.115	0.000	0.000	0.000	0.000	0.000	0.000	0.000	0.000	0.000	0.000	0.000	0.000
	235	0.137	-0.004	0.000	0.000	-0.022	0.000	0.000	0.000	0.000	-0.007	-0.027	0.091	0.000	-0.029	-0.055	0.014	0.000	-0.007	-0.004	-0.044
	236	0.000	-0.015	0.000	-0.007	-0.004	-0.011	0.000	0.000	0.000	0.244	-0.015	-0.004	-0.011	-0.025	0.030	0.000	0.000	0.000	-0.133	-0.004
Helix 7	237	0.000	0.000	0.000	0.000	-0.011	-0.011	-0.033	0.000	0.105	0.000	0.000	0.000	0.000	0.000	-0.004	0.000	0.000	0.000	0.000	0.000
	238	0.075	0.000	-0.002	0.109	-0.004	-0.053	-0.004	0.000	-0.004	0.000	-0.007	-0.011	-0.011	0.000	-0.004	-0.011	0.000	-0.031	0.000	0.000
	239	-0.007	0.000	-0.004	0.031	0.000	-0.076	0.187	0.000	-0.140	0.000	0.000	0.068	-0.020	-0.007	0.013	-0.018	0.000	0.000	0.000	0.018
	240	-0.011	0.069	0.000	0.000	0.000	-0.011	0.000	0.000	0.000	0.000	0.000	0.000	0.000	0.000	0.000	-0.004	0.000	0.000	0.000	0.000
	241	0.000	-0.004	0.000	0.000	0.000	0.000	0.000	0.000	0.000	0.000	0.051	0.000	0.000	0.000	0.000	0.000	0.000	0.000	0.000	-0.004

-0.360 0.000 0.360

Δ Amino acid frequency per HeR

Supplementary Fig. 3. Difference of amino acid frequency per HeR in classified 10 groups compared to the non-co-transcription group (*helR* alone).

The amino acids of each HeR in the classified ten groups and no co-transcription group at the residue positions in OcHeR are counted and subsequently normarlized by the number of HeRs in the those groups, that is, the amino acid frequency per HeR. The difference of amino acid frequency per HeR are indicated by cyan (decrease) and pink (increase) colors. All data are analyzed from data in Supplementary Data 12 and 19.

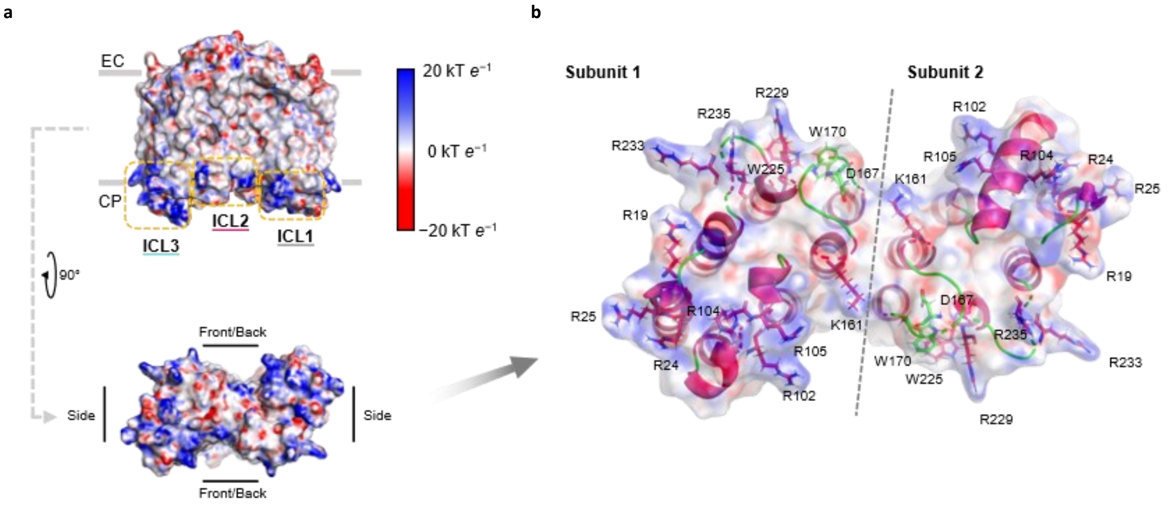

Supplementary Fig. 4. Dominant charged residues in HeRs.

a,**b** Blue and red surfaces in the electrostatic potential distribution of OcHeR represent positively and negatively charged residues, respectively. Red and blue correspond to potentials of 20 kT e^{-1} and −20 kT e^{-1}, respectively. **a** Upper and lower figures are viewed from membrane and cytoplasm angles, respectively, and yellow dotted rectangles indicate ICLs. **b** Enlargement of view from the cytoplasm angle. The OcHeR homodimer is distinguished by subunit 1 and subunit 2 using a dotted gray line.

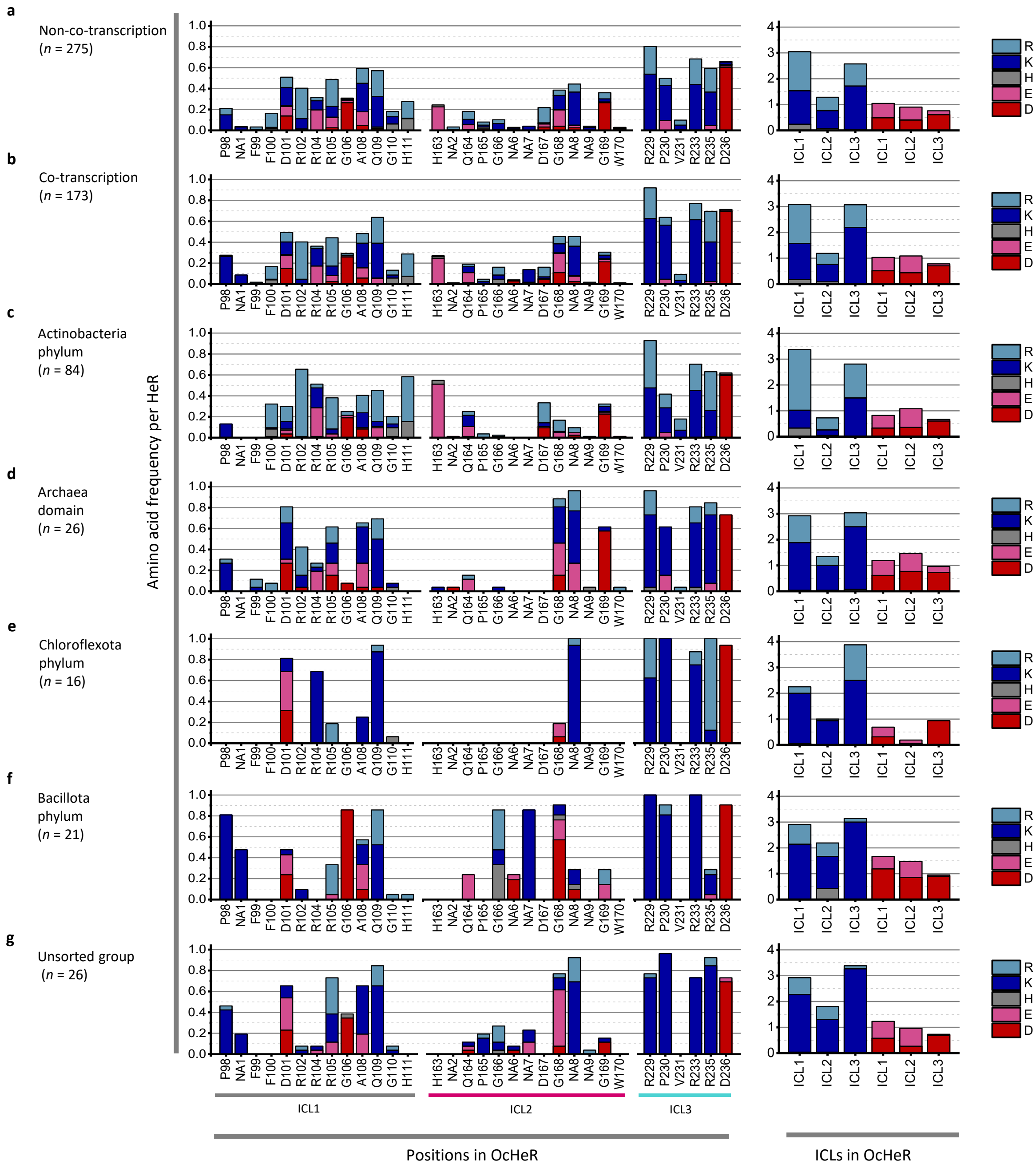

Supplementary Fig. 5. Analyzing charged residues among HeRs.

HeR sequences were analyzed from phylogenetic trees between the HeRs (total: 448 HeR sequences) in organisms based on steps 1–4 in Supplementary Fig. 1b and data in Supplementary Data 11–18. The charged residues of each HeR at the residue positions in the ICLs of OcHeR were counted and subsequently normalized by total HeRs, which is the indicated amino acid frequency per HeR (e.g. a value of 1 means that one HeR contains one of those residue). **a,b** Charged residues in the non-co-transcription (n = 275) and co-transcription *helR* class (n = 173) in the organisms. **c–g** Charged residues in Actinobacteria phylum (n = 84), Archaea domain (n = 26), Chloroflexota phylum (n = 16), Bacillota phylum (n = 21), and unsorted group (n = 26), involved in co-transcription *helR* class.

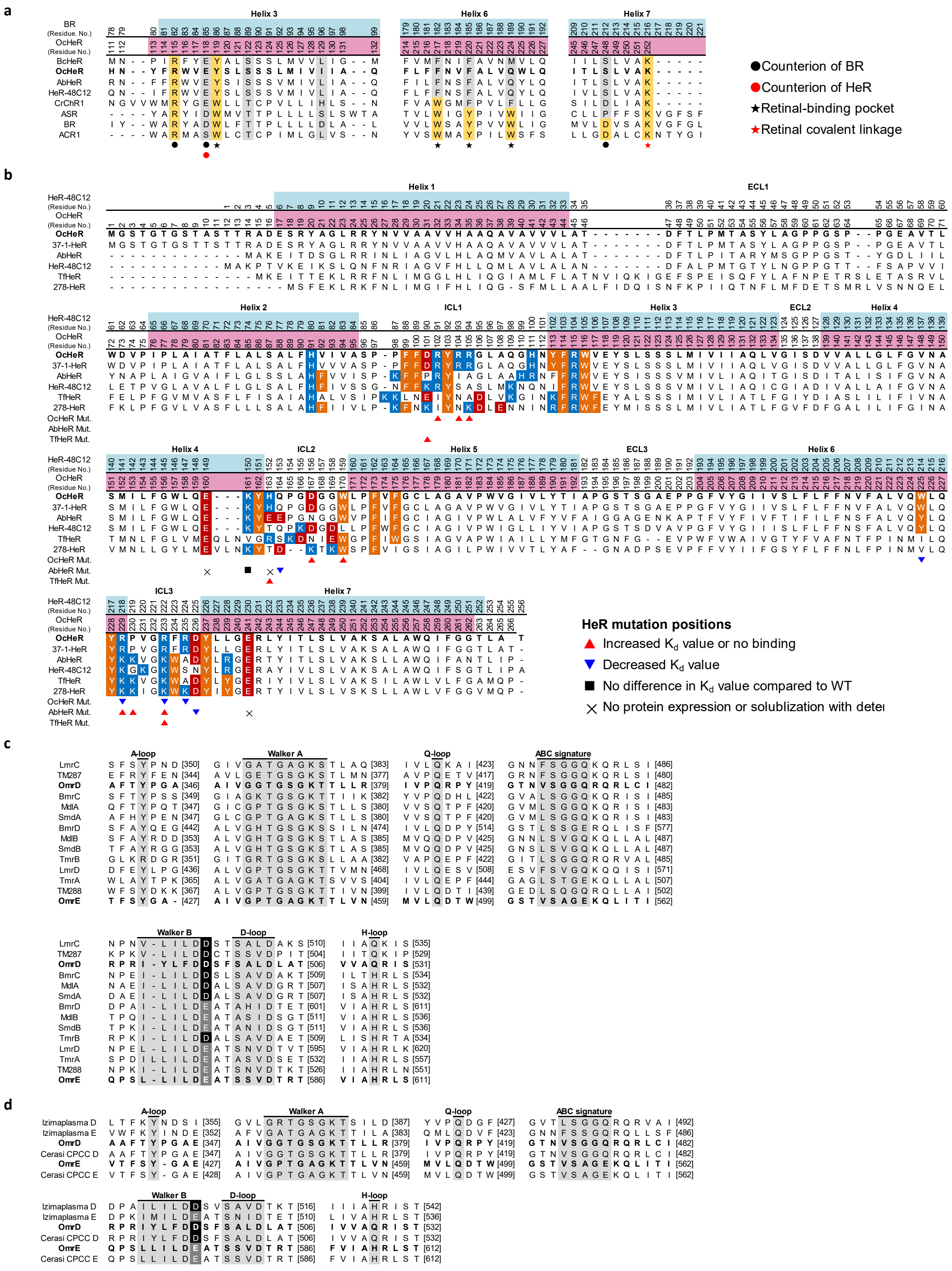

Supplementary Fig. 6. Protein sequence alignments of rhodopsins and ABCTs.

Multiple sequence alignments of microbial rhodopsins, HeRs, and ABCTs were performed using MUSCLE. The helices of OcHeR are based on the 3D structure (PDB code: 6SU4), which was modeled using a protein structure homology-modeling server (Swiss-Model). **a** Alignment of microbial rhodopsins and HeRs. BcHeR, *Bellilinea caldifistulae* HeR; CrChR1, *Chlamydomonas reinhardtii* channelrhodopsin (ChR) 1; ASR, *Anabaena* spp. PCC7120, sensory rhodopsin; BR, *Halobacterium salinarum* bacteriorhodopsin; ACR1, *Guillardia theta*

anionrhodopsin. **b** Alignment of HeRs. 37-1-HeR, *Ornithinimicrobium cerasi* strain CPCC 203383 HeR; AbHeR, *Actinobacteria bacterium* IMCC26103 HeR; HeR-48C12, *Actinobacterium* clone fosmid 48C12 HeR; TfHeR, *Trichococcus flocculiformis* HeR; 278-HeR, *Candidatus Izimaplasma* sp. ZiA1 HeR. Point mutation positions in this and previous studies are marked. **c** Alignment of OmrD, OmrE, and multidrug ABCTs. **d** Alignment of HeRs in the group of ABCT-containing operons. **c**,**d** Highly conserved motifs for ATP binding/hydrolysis in the NBD of ABCT are highlighted in light gray. Canonical (Glu) and non-canonical (Asp) residues are highlighted in dark gray and black, respectively. All protein sequences are available with Supplementary Data 20 and Source data.

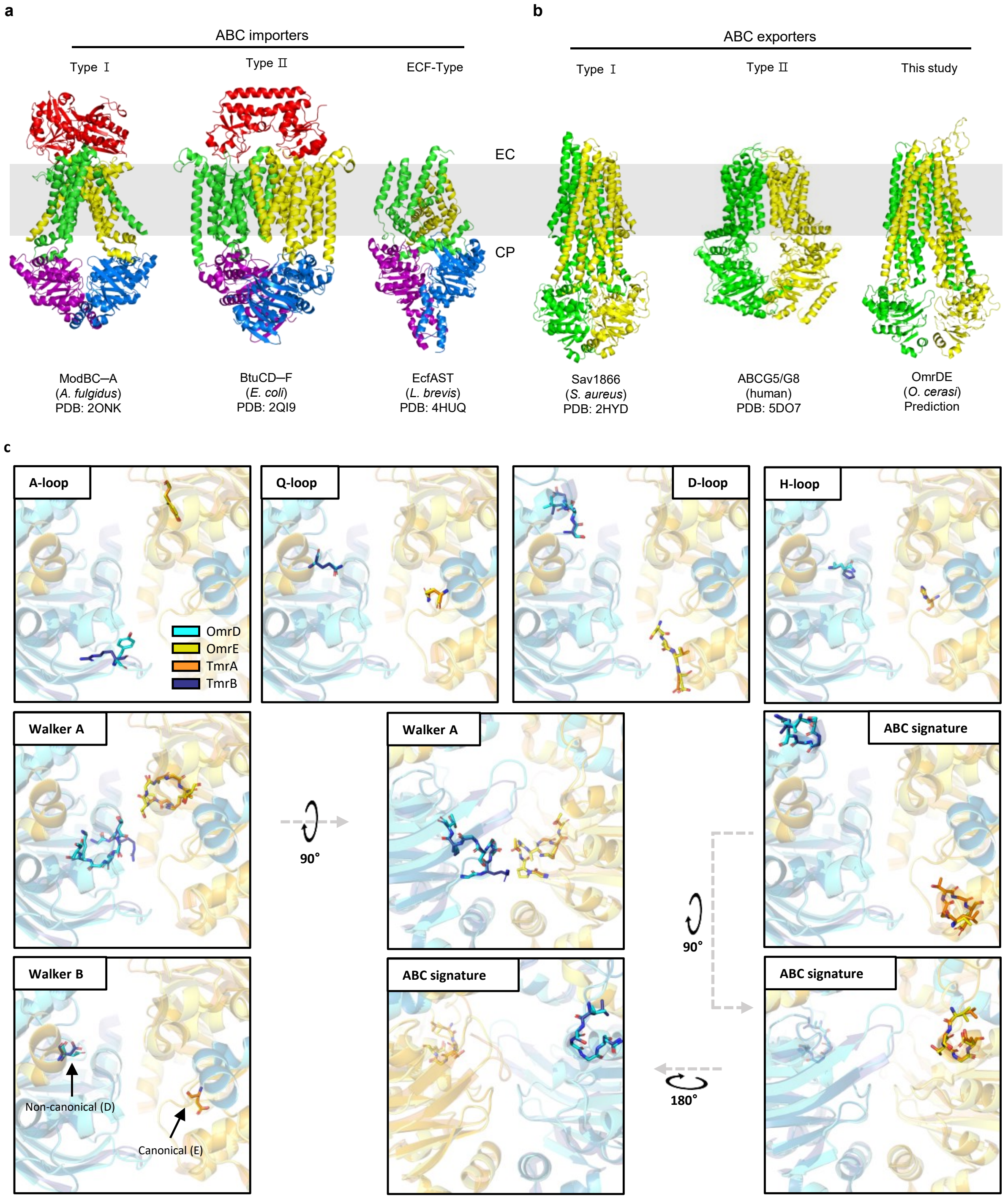

Supplementary Fig. 7. Analysis of crystal structures of ABCTs.

a ABC importers are shown as follows: yellow and green, TMDs; red, periplasmic substrate-binding proteins; purple and blue, NBDs. **b** TMDs of the ABC exporters are shown in yellow and green, and the TMDs and NBDs are fused. The modeled OmrDE heterodimer was performed using a protein structure homology-modeling server (Swiss-Model) based on TmrAB (PDB code: 6RAF). Extracellular and cytoplasmic sides are labeled EC and CP, respectively. **c** Comparison of protein structures between OmrDE (predicted by TmrAB as a template) and TmrAB (only TMDs and NBDs are indicated). The protein structures are colored: OmrD, cyan; OmrE, yellow; TmrA, orange; TmrB, navy. The functional important motifs in

OmrDE and TmrAB are indicated by sticked residues.

a

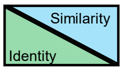

	LmrC	TM287	**OmrD**	BmrC	MdlA	SmdA	MsbA	BmrD	MdlB	SmdB	TmrB	Sav1866	LmrD	TmrA	TM288	**OmrE**
LmrC	100%	71%	**69%**	63%	63%	62%	64%	63%	64%	61%	66%	66%	65%	63%	62%	**64%**
TM287	38%	100%	**73%**	66%	65%	66%	66%	62%	64%	64%	67%	65%	66%	68%	68%	**66%**
**OmrD**	**33%**	**40%**	**100%**	**67%**	**64%**	**65%**	**67%**	**63%**	**66%**	**66%**	**65%**	**67%**	**64%**	**67%**	**65%**	**69%**
BmrC	27%	29%	**27%**	100%	70%	70%	64%	63%	62%	62%	68%	66%	60%	64%	65%	**65%**
MdlA	24%	27%	**26%**	41%	100%	91%	64%	60%	62%	62%	67%	65%	62%	65%	61%	**61%**
SmdA	25%	27%	**26%**	41%	79%	100%	65%	60%	62%	62%	67%	67%	63%	65%	62%	**62%**
MsbA	24%	26%	**27%**	28%	28%	29%	100%	65%	66%	66%	64%	69%	66%	69%	67%	**66%**
BmrD	26%	29%	**29%**	29%	24%	24%	28%	100%	70%	70%	62%	67%	58%	67%	64%	**62%**
MdlB	24%	26%	**27%**	25%	24%	25%	29%	38%	100%	92%	64%	66%	63%	66%	64%	**68%**
SmdB	21%	25%	**27%**	24%	26%	26%	29%	36%	79%	100%	64%	65%	63%	67%	66%	**68%**
TmrB	25%	30%	**27%**	34%	33%	33%	28%	27%	28%	26%	100%	66%	62%	70%	65%	**61%**
Sav1866	28%	29%	**28%**	31%	29%	31%	33%	31%	30%	29%	28%	100%	66%	70%	68%	**66%**
LmrD	23%	30%	**25%**	25%	26%	26%	27%	27%	25%	26%	23%	29%	100%	64%	69%	**63%**
TmrA	26%	30%	**31%**	28%	29%	29%	30%	33%	30%	30%	34%	34%	30%	100%	72%	**69%**
TM288	26%	33%	**30%**	29%	28%	27%	33%	32%	29%	30%	29%	35%	36%	37%	100%	**73%**
**OmrE**	**25%**	**28%**	**29%**	**29%**	**28%**	**30%**	**29%**	**27%**	**31%**	**30%**	**26%**	**30%**	**30%**	**33%**	**38%**	**100%**

b

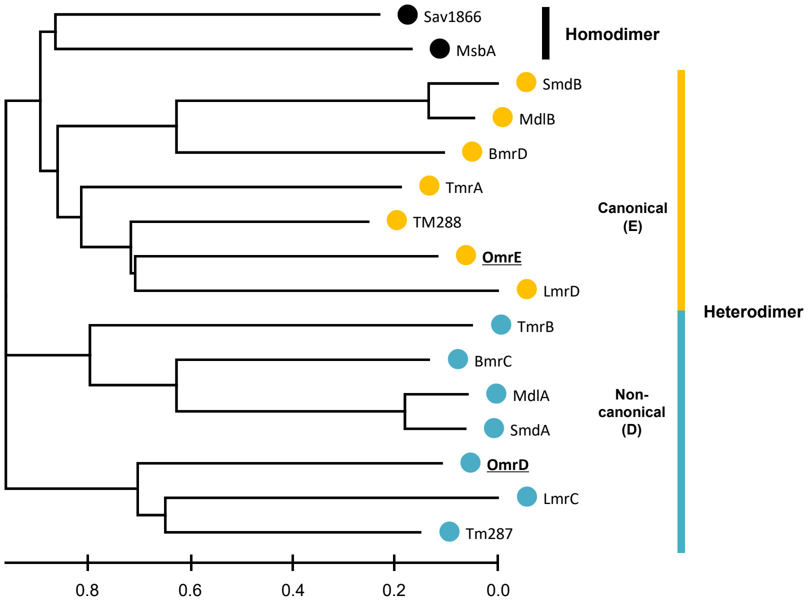

Supplementary Fig. 8. Comparison of the multidrug transporters, OmrD, and OmrE.

a,**b** Sixteen ABCTs were used to analyze the protein sequence alignments and evolutionary relationships among multidrug transporters. **a** Pairwise sequence identities and similarities from multiple sequence alignments are highlighted in green and sky blue, respectively. **b** The phylogenetic tree of ABCT was inferred using the neighbor-joining method. Homo- and heterodimeric clades are indicated using different colored bars and circles. Canonical and non-canonical structures in the heterodimer clade are indicated by yellow and sky-blue bars and circles, respectively.

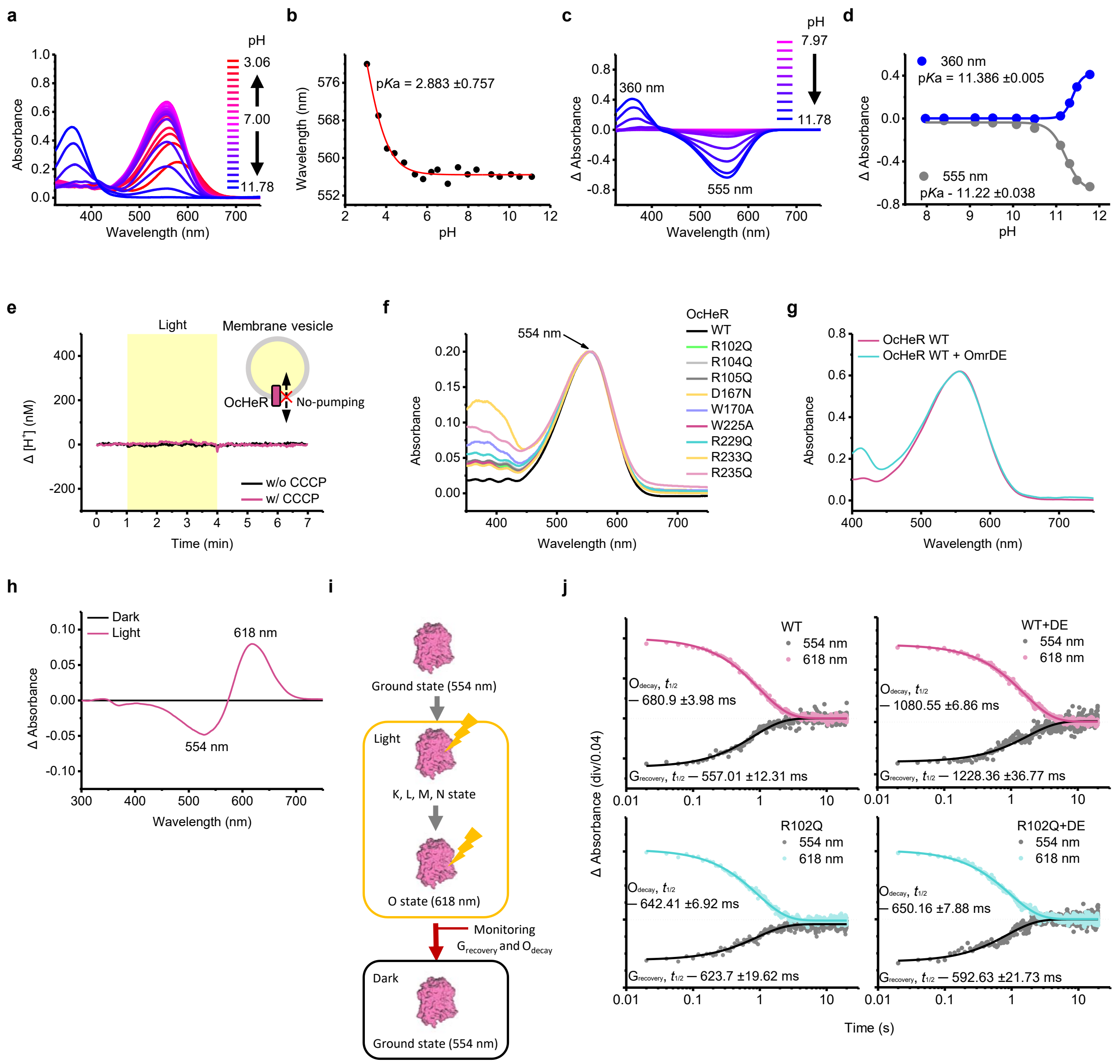

Supplementary Fig. 9. Photochemical characteristics of OcHeR.

a Absorption spectra of OcHeR WT at different pH values. **b** The spectral red-shift of the λ_{max} of OcHeR at pH 3.06–11.78. The p*K*a value of counterion (presumably E118) of OcHeR based on the λ_{max} of OcHeR WT at different pH values was estimated using the Henderson–Hasselbalch equation. **c** Difference absorption spectra of OcHeR WT at an alkaline pH. The absorption changes and the deprotonated form of retinal Schiff base showed at 555 and 360 nm, respectively. The p*K*a values of the absorption changes and the retinal Schiff Base linked to K252 of OcHeR based on difference absorption were estimated using the Henderson–Hasselbalch equation. **b,d** The p*K*a values are expressed as the value ± error. **e** Light-induced H^+ movement assay of OcHeR WT membrane vesicles was performed in the absence and presence of light (1–4 min, yellow space). Pink and black lines indicate with and without CCCP, respectively. **f** Absorption spectra of OcHeR WT and the mutants. **g** The absorption spectra of OcHeR WT and OcHeR WT with OmrDE after binding for 1 h. **h** Delta absorption spectra of OcHeR WT in the absence (dark) and presence (light) of light, which showed two peaks [554 nm = G (ground) state; 618 nm = O state]. **i** Schematic experimental procedures of a photocycle

measurement. In the absence of light, the $G_{recovery}$ and O_{decay} based on the two wavelengths were monitored every 20 ms after illumination to excite rhodopsin for 10 s. **j** Photocycles of OcHeR with and without OmrDE were recorded at 618 nm (O state) and 554 nm (G state); half-life ($t_{1/2}$) values through data analysis were quantified by exponential decay as non-linear fitted lines.

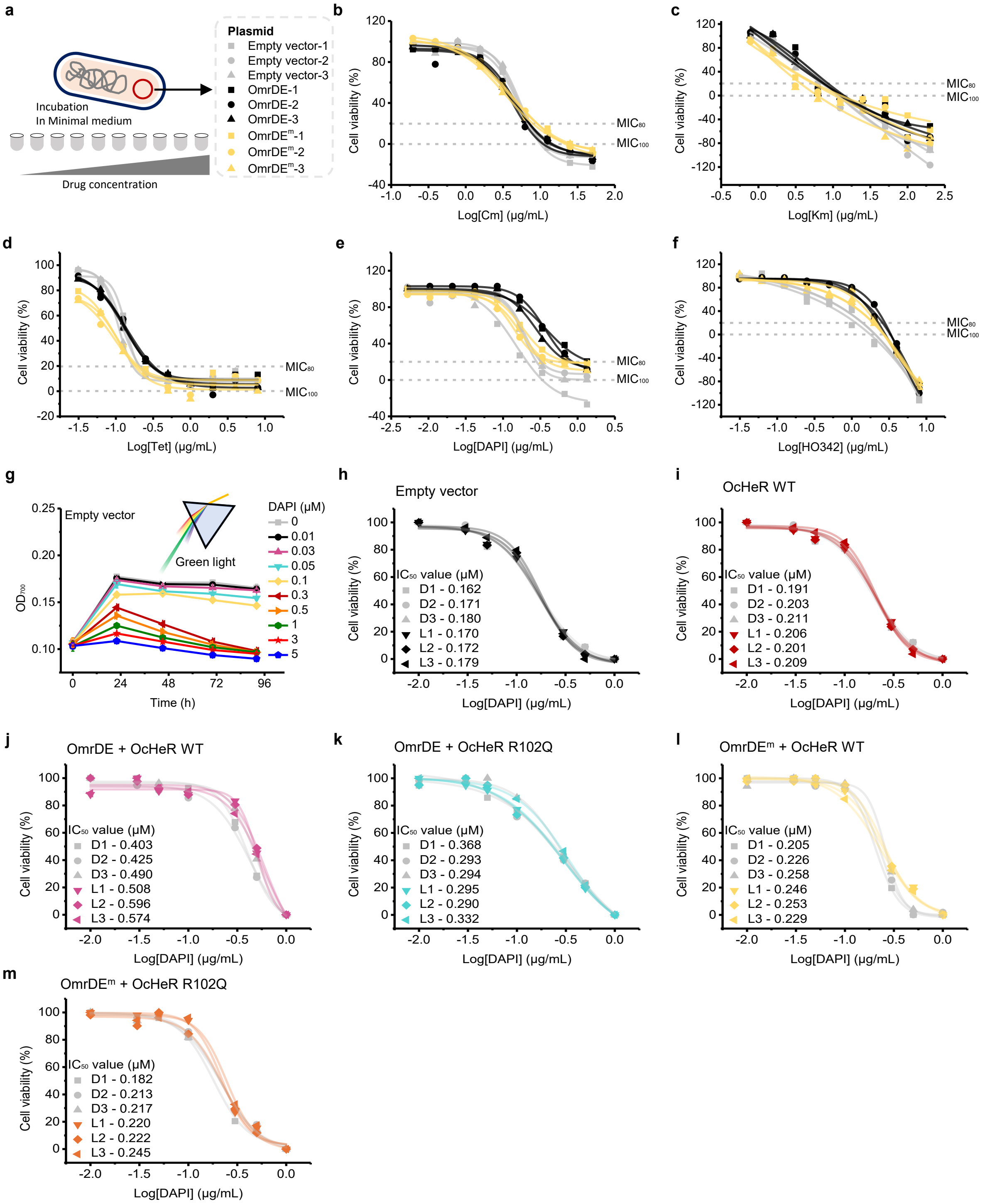

Supplementary Fig. 10. Multidrug resistance test of *E. coli* N43 harboring plasmids.

a *E. coli* N43 strains harboring plasmids overexpressing none, OmrDE, or OmrDE^{m} were cultured in LB medium and washed three times with MOPS minimal medium. *E. coli* cells were diluted with MOPS minimal medium and treated with different concentrations of various drugs (B to F). **a–f** An independent experimental group (n = 3) is indicated by square, circle, triangle symbols. (**b**) Cm, chloramphenicol; (**c**) Km, kanamycin; (**d**) Tet, tetracycline; (**e**) DAPI, 4′,6-diamidino-2-phenylindole; and (**f**) HO342, Hoechst 33342. *E. coli* growth was determined by measuring the OD_{700} at 0 and 45 h, and cell viability was calculated by subtracting the OD_{700}

at 0 from that at 45 h. The MIC_{100} dotted line represents equal OD_{700} values at 0 and 45 h. The MIC_{80} dotted line indicates that the drug can inhibit cell growth by up to 80% compared with drug-free cells. MIC_{80} values were calculated from the non-linear curves using the DoseResp equation. **g–m** *E. coli* N43 strains harboring plasmids overexpressing none, OcHeR WT, OmrDE+OcHeR WT, OmrDE+OcHeR R102Q, $OmrDE^{m}$+OcHeR WT, or $OmrDE^{m}$+OcHeR R102Q were cultured in LB medium and washed three times with MOPS minimal medium. *E. coli* cells were diluted with MOPS minimal medium and treated with different concentrations of DAPI. **g** Growth curves of *E. coli* treated with different concentrations of DAPI were measured at OD_{700} in the presence of light (light, 532 nm). Data are presented as mean value ± SD. **h–m** *E. coli* growth in the absence (dark, D) and presence of light (light, L, 532 nm) was measured by measuring OD_{700} at 0 and 45 h. Cell viability was calculated by subtracting the OD_{700} at 0 h from that at 45 h. IC_{50} values were fitted using the DoseResp equation. In (**g–m**), measurements were conducted in an independent experimental group (n = 3).

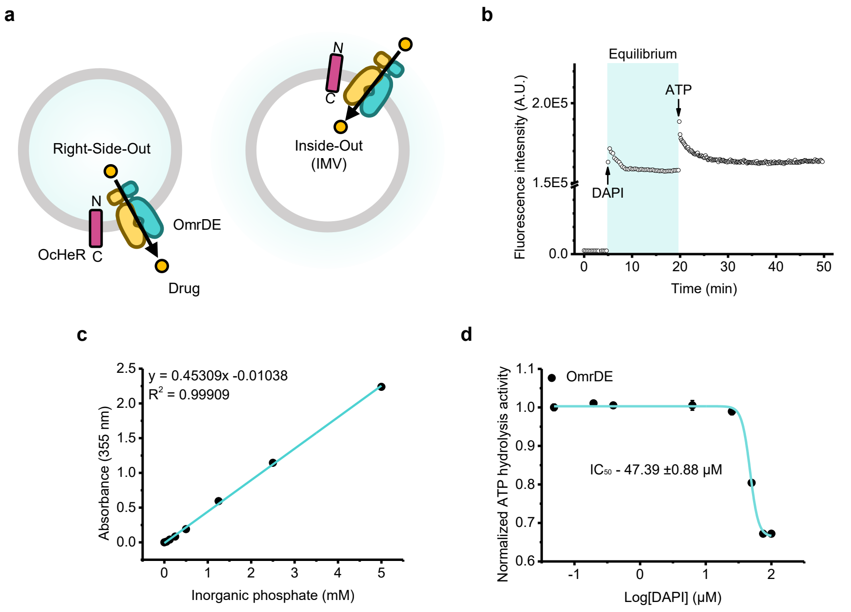

Supplementary Fig. 11. Determining OmrDE activity *in vitro*.

a Schematics of right-side-out and inside-out membrane vesicles containing membrane proteins. Orientation of the drug translocation is indicated using a black arrow. **b** Drug transport assay in IMV-containing OmrDE shows time-tracing fluorescence intensity after treatment with DAPI and ATP at each timepoint: 0–5 min, IMV; 5–20 min, IMV + DAPI; 20–50 min, IMV + DAPI + ATP. **c** P_i standard curves using different P_i concentrations were fitted to a linear equation; the equation and R-squared values are indicated. **d** ATP hydrolysis assay of the NBDs in purified OmrDE with different DAPI concentrations. IC_{50} values were fitted using the DoseResp function, as described in the Methods section. **c**,**d** Measurements were conducted in an independent experimental group (n = 3). Data were presented as value means ± SD.

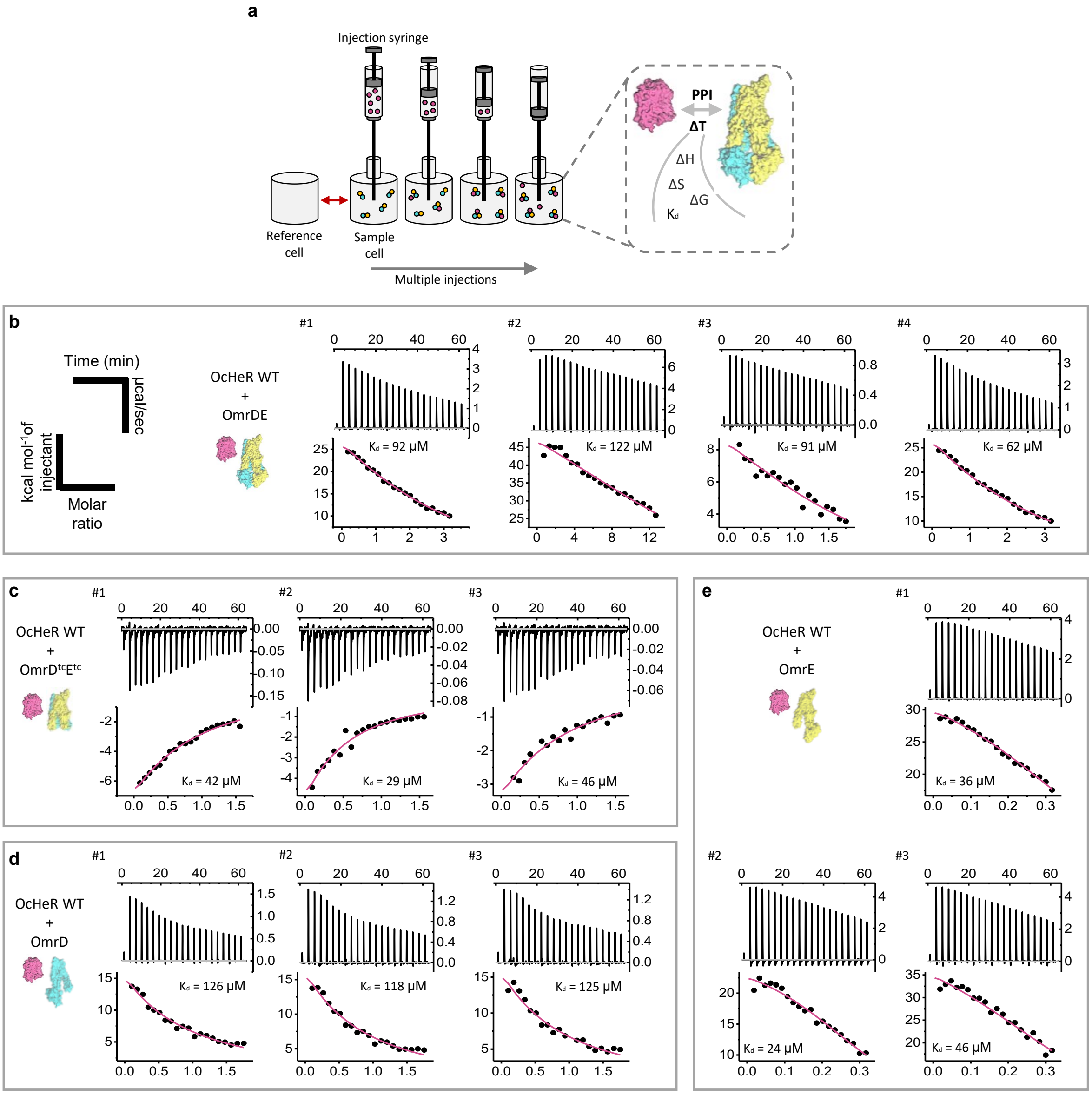

Supplementary Fig. 12. Binding affinities of the OcHeR WT for OmrDE.

a Schematic experiments of ITC analysis. OcHeR in the injection syringe was continuously injected into OmrDE in the sample cell. The thermal change (ΔT) between the reference cell and sample cell determines binding affinity and thermodynamic parameters. **b**–**e** Binding affinities of the OcHeR WT for OmrDE were determined using a one binding site model through ITC analysis. The OcHeR WT were continuously added to OmrDE. The upper and lower panels represent raw data and enthalpy changes per mol, respectively. Non-linear curves in the lower panels represent the best-fit curve. Measurements were conducted in an independent experimental group (n = 3 or 4). Each the K_d value is indicated in figures.

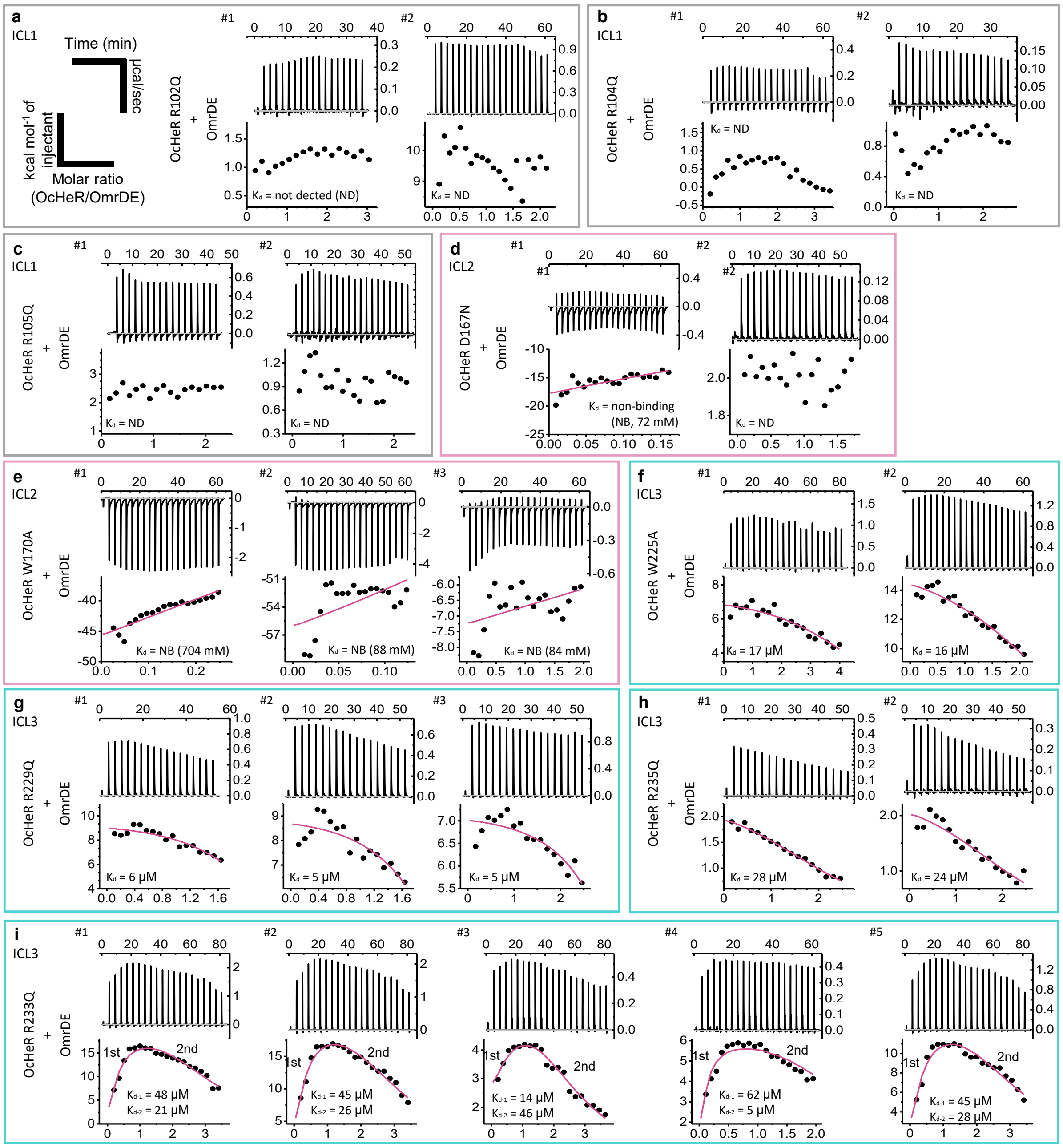

Supplementary Fig. 13. Binding affinities of the OcHeR mutants for OmrDE.

a–**i** Binding affinities of the OcHeR mutants for OmrDE were determined using a one binding site model (**a**–**h**) and a sequential binding site model (**i**) through ITC analysis. The OcHeR mutants were continuously added to OmrDE. The upper and lower panels represent raw data and enthalpy changes per mol, respectively. Non-linear curves in the lower panels represent the best-fit curve. **d,e** D167N and W170A mutants with a large difference in the K_d value compared with OcHeR WT for OmrDE were considered non-binding. Measurements were conducted in an independent experimental group (*n* = 2 to 5).

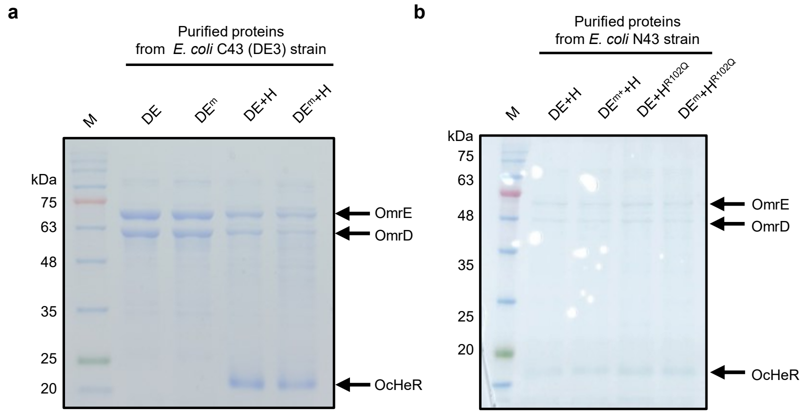

Supplementary Fig. 14. Sodium dodecyl sulphate polyacrylamide gel electrophoresis (SDS–PAGE) analysis of purified membrane proteins.

Abbreviations: M, protein marker; DE, OmrDE; DE^{m}, $OmrDE^{E577Q}$; H, OcHeR WT; H^{R102Q}, OcHeR R102Q. The molecular weight of the purified membrane protein containing a hexahistidine tag was calculated using amino acid sequences: OcHeR, 29.43 kDa; OmrD, 62.86 kDa; and OmrE, 72.09 kDa. **a,b** Purified membrane proteins (OmrDE and OcHeR) from *E. coli* C43 (DE3) and *E. coli* N43 were subjected to SDS-PAGE using stacking (12%) and separating (5%) Tris-Glycine gels.

Supplementary Table 1. Drug sensitivity of *E. coli* N43 strain harboring plasmids.

	**MIC_{80} values (µg/mL)**		
**Drugs**	**Empty vector**	**OmrDE**	**$OmrDE^m$**
Chloramphenicol	6.986 ±0.151	6.807 ±0.435	8.647 ±0.545
Kanamycin	7.541 ±0.368	7.904 ±0.517	4.947 ±0.906
Tetracycline	0.194 ±0.019	0.306 ±0.010	0.189 ±0.009
DAPI	0.272 ±0.046	1.047 ±0.335	0.587 ±0.165
Hoechst 33342	1.302 ±0.122	2.543 ±0.210	1.997 ±0.087

MIC_{80} values are estimated from the non-linear curves data in Supplementary Figs. 10b–f.

Measurements were conducted in an independent experimental group (n = 3).

Values are presented as mean value ± SD.

Supplementary Table 2. Oligomers used to amplify DNA fragments during plasmid preparation.

Oligomer	Sequence (5'→3')
F1	AGATCTCCCCGACACCCGCCA
R1	ACTAGTGCTAGCTCTCCTTCTTAAAGTTAAAC
F2	GCTAGCACTAGTGTTTAAACGGTCTCCAGCTTGG
R2	AGATCTGATGCCTGGCAGTTTATGGCG
F3	GCTAGCATGCATCACCATCACCACCAT
R3	GTTTAAACTTAGGTTGCAAGCGTG
F4	GGAGATATCATATGTCTACTCGCG
R4	CTCCTCGAGATCTGGGCGTTGTTGTTC
F5	AGAGAATTCATGCTTACCCG
R5	AGACATATGATATCTCCTTCTTAAAGTTAAACAAATTAGTGGTGGTG GTGGTGATGGCCCACTGACGACTCAGT
F6	AACAATTCCCCTCTAGAAATAATTTTG
R6	GACTCCTCGAGTTAGTCGGCATCGACCTGTAC

The recognition sites for restriction enzymes are underlined.

Supplementary Table 3. Plasmids used in this study.

Plasmid	Relevant characteristics for protein expression
pET21b+ (empty)	pT7::
pET21b-DE	pT7::OmrD::OmrE
pET21b-D	pT7::OmrD
pET21b-E	pT7::OmrE
pACHA-E	pT7::OmrE, p15A origin, 3xHA (hemagglutinin)-tag at N-terminus
pET21b-$D^{tc}E^{tc}$	pT7::OmrDΔ320–577::OmrEΔ402–661
pET21b-DEm	pT7::OmrD::OmrE D577Q
pET21b-H	pT7::OcHeR WT
pET21b-H^{m} or H^{R102Q}	pT7::OcHeR R102Q
pET21b-H^{R104Q}	pT7::OcHeR R104Q
pET21b-H^{R105Q}	pT7::OcHeR R105Q
pET21b-H^{D167N}	pT7::OcHeR D167N
pET21b-H^{W170A}	pT7::OcHeR W170A
pET21b-H^{W225A}	pT7::OcHeR W225A
pET21b-H^{W229A}	pT7::OcHeR W229A
pET21b-H^{R233Q}	pT7::OcHeR R233Q
pET21b-H^{R235Q}	pT7::OcHeR R235Q
pET21b-DEH	pT7::OmrD::OmrE, pBad::OcHeR WT
pET21b-DEH^{m}	pT7::OmrD::OmrE, pBad::OcHeR R102Q
pKA001-DEH	pLac::OmrD::OmrE, pBad::OcHeR WT
pKA001-DEH^{m}	pLac::OmrD::OmrE, pBad::OcHeR R102Q
pKA001-$DE^{m}H$	pLac::OmrD::OmrE D577Q, pBad::OcHeR WT
pKA001-$DE^{m}H^{m}$	pLac::OmrD::OmrE D577Q, pBad::OcHeR R102Q

References

1. Locher, K. P. Mechanistic diversity in ATP-binding cassette (ABC) transporters. *Nat Struct Mol Biol* **23**, 487–493 (2016).
2. Hofmann, S. *et al.* Conformation space of a heterodimeric ABC exporter under turnover conditions. *Nature* **571**, 580–583 (2019).
3. Bienert, S. *et al.* The SWISS-MODEL Repository—new features and functionality. *Nucleic Acids Res* **45**, D313–D319 (2017).
4. Guex, N., Peitsch, M. C. & Schwede, T. Automated comparative protein structure modeling with SWISS-MODEL and Swiss-PdbViewer: A historical perspective. *Electrophoresis* **30**, S162–S173 (2009).
5. Ladbury, J. E., Klebe, G. & Freire, E. Adding calorimetric data to decision making in lead discovery: a hot tip. *Nat Rev Drug Discov* **9**, 23–27 (2010).
6. Wilson, D. F. Regulation of cellular metabolism: programming and maintaining metabolic homeostasis. *Journal of Applied Physiology* **115**, 1583–1588 (2013).
7. Shimizu, K. Metabolic Regulation of a Bacterial Cell System with Emphasis on Escherichia coli Metabolism. *ISRN Biochem* **2013**, 645983 (2013).
8. Lee, E.-H. *et al.* Enhancement of enzyme activity and stability by poly(γ-glutamic acid). *Polym J* **42**, 818–822 (2010).
9. Chang, C. C., Few, L. L., Konrad, M. & Too, W. C. S. Phosphorylation of Human Choline Kinase Beta by Protein Kinase A: Its Impact on Activity and Inhibition. *PLoS ONE* **11**, e0154702 (2016).
10. Cho, S.-G. *et al.* Heliorhodopsin binds and regulates glutamine synthetase activity. *PLoS Biology* **20**, e3001817 (2022).
11. Cho, S.-G. *et al.* Discovery of a new light-driven Li^+/Na^+-pumping rhodopsin with DTG motif. *J Photochem Photobiol B: Biol* **223**, 112285 (2021).

12. Díaz-Cárdenas, C. *et al.* Microbial diversity of saline environments: searching for cytotoxic activities. *AMB Express* **7**, 223 (2017).
