# Supplementary material for: Heliorhodopsin-mediated light-modulation of ABC transporter
Source: Nat Commun. 2024 May 21;15:4306. doi: 10.1038/s41467-024-48650-1 (PMC11109279; doi:10.1038/s41467-024-48650-1)
Supplement: Supplementary file 3 — Description of Additional Supplementary Files [file 41467_2024_48650_MOESM3_ESM.pdf]

Description of Additional Supplementary Files

File Name: Supplementary Data 1-10
Description:

1. Operons in which *helR* and ABC transporter-encoding genes are co-transcribed.
2. Operons in which *helR* and glutamine synthetase-encoding genes are co-transcribed.
3. Operons in which *helR* and photolyase-encoding genes are co-transcribed.
4. Operons in which *helR* and DUF2177-encoding genes are co-transcribed.
5. Operons in which *helR* and alpha/beta hydrolase-encoding genes are co-transcribed.
6. Operons in which *helR* and oxidoreductase-encoding genes are co-transcribed.
7. Operons in which *helR* and NAD+ synthase-encoding genes are co-transcribed.
8. Operons in which *helR* and DUF2238-encoding genes are co-transcribed.
9. Operons in which *helR* and TerC protein-encoding genes are co-transcribed.
10. Operons in which *helR* and transcriptional regulator-encoding genes are co-transcribed.

File Name: Supplementary Data 11-18
Description:

11. Alignment of total HeR sequences.
12. Alignment of HeR sequences in non-co-transcription from total *helR*.
13. Alignment of HeR sequences in co-transcription from total *helR*.
14. Alignment of HeR sequences in co-transcription from Actinobacteria phylum.
15. Alignment of HeR sequences in co-transcription from Archaea domain.
16. Alignment of HeR sequences in co-transcription from Chloroflexota phylum.
17. Alignment of HeR sequences in co-transcription from Bacillota phylum.
18. Alignment of HeR sequences in co-transcription from unsorted group.

File Name: Supplementary Data 19
Description: Alignment of HeR sequences in classified 10 groups from total helR.

File Name: Supplementary Data 20
Description: Rhodopsin database for phylogenetic tree of heliorhodopsins and microbial rhodopsins.
