# Supplementary material for: Heliorhodopsin-mediated light-modulation of ABC transporter
Source: Nat Commun. 2024 May 21;15:4306. doi: 10.1038/s41467-024-48650-1 (PMC11109279; doi:10.1038/s41467-024-48650-1)
Supplement: Supplementary file 8 — Reporting Summary [file 41467_2024_48650_MOESM8_ESM.pdf]

nature portfolio

Corresponding author(s): Kwang-Hwan Jung

Last updated by author(s): Mar 31, 2024

Reporting Summary

Nature Portfolio wishes to improve the reproducibility of the work that we publish. This form provides structure for consistency and transparency in reporting. For further information on Nature Portfolio policies, see our Editorial Policies and the Editorial Policy Checklist.

Statistics

For all statistical analyses, confirm that the following items are present in the figure legend, table legend, main text, or Methods section.

n/a	Confirmed	
☐	☒	The exact sample size (*n*) for each experimental group/condition, given as a discrete number and unit of measurement
☐	☒	A statement on whether measurements were taken from distinct samples or whether the same sample was measured repeatedly
☐	☒	The statistical test(s) used AND whether they are one- or two-sided<br>*Only common tests should be described solely by name; describe more complex techniques in the Methods section.*
☒	☐	A description of all covariates tested
☒	☐	A description of any assumptions or corrections, such as tests of normality and adjustment for multiple comparisons
☐	☒	A full description of the statistical parameters including central tendency (e.g. means) or other basic estimates (e.g. regression coefficient) AND variation (e.g. standard deviation) or associated estimates of uncertainty (e.g. confidence intervals)
☐	☒	For null hypothesis testing, the test statistic (e.g. *F*, *t*, *r*) with confidence intervals, effect sizes, degrees of freedom and *P* value noted<br>*Give P values as exact values whenever suitable.*
☒	☐	For Bayesian analysis, information on the choice of priors and Markov chain Monte Carlo settings
☒	☐	For hierarchical and complex designs, identification of the appropriate level for tests and full reporting of outcomes
☒	☐	Estimates of effect sizes (e.g. Cohen's *d*, Pearson's *r*), indicating how they were calculated

Our web collection on statistics for biologists contains articles on many of the points above.

Software and code

Policy information about availability of computer code

Data collection	BLAST+ v2.14 in the National Center for Biotechnology Information (all genes and genomes)
Data analysis	OriginPro v9.0 (dose–response , exponential decay, and statistical analyses), Origin 7.0 SR4 (ITC analysis), Multiple Sequence Alignment (MUSCLE in EMBL), Promoter prediction tools (phSITE, Softberry, BDGP, and SAPPHIRE), Swiss-Model (protein structure homology-modeling server), PyMOL v2.5.5 (molecular graphics software), CHARMM-GUI v3.8 (electrostatic potential distribution analysis), ClusPro v2.0 (Protein–protein docking simulation)

For manuscripts utilizing custom algorithms or software that are central to the research but not yet described in published literature, software must be made available to editors and reviewers. We strongly encourage code deposition in a community repository (e.g. GitHub). See the Nature Portfolio guidelines for submitting code & software for further information.

Data

Policy information about availability of data

All manuscripts must include a data availability statement. This statement should provide the following information, where applicable:

- Accession codes, unique identifiers, or web links for publicly available datasets
- A description of any restrictions on data availability
- For clinical datasets or third party data, please ensure that the statement adheres to our policy

The genome of the O. cerasi strain USBA17B2 is collected in the NCBI under accession number OBQK01000028 and the Joint Genome Institute under project ID

1094766. Genes encoding protein used to recombinant protein in this study are available in NCBI under accession numbers SOC58301 (OcHeR), SOC58303 (OmrE), and SOC58305 (OmrD). The template protein crystal structures for predicting the protein crystal structure of OcHeR and OmrDE are available in Protein Data Bank under PDB codes 6SU4 (HeR-48C12) and 6RAF (TmrAB inward-facing conformation). All data used in this study are available in Methods, Supplementary information, Supplementary data, and Source data. Source data and Supplementary data are provided with this paper.

Research involving human participants, their data, or biological material

Policy information about studies with human participants or human data. See also policy information about sex, gender (identity/presentation), and sexual orientation and race, ethnicity and racism.

Reporting on sex and gender	Not applicable for this study.
Reporting on race, ethnicity, or other socially relevant groupings	Not applicable for this study.
Population characteristics	Not applicable for this study.
Recruitment	Not applicable for this study.
Ethics oversight	Not applicable for this study.

Note that full information on the approval of the study protocol must also be provided in the manuscript.

Field-specific reporting

Please select the one below that is the best fit for your research. If you are not sure, read the appropriate sections before making your selection.

☒ Life sciences ☐ Behavioural & social sciences ☐ Ecological, evolutionary & environmental sciences

For a reference copy of the document with all sections, see nature.com/documents/nr-reporting-summary-flat.pdf

Life sciences study design

All studies must disclose on these points even when the disclosure is negative.

Sample size	Because each size of Escherichia coli and proteins could not be calculated, the sample sizes were not calculated, and the amounts of E. coli and proteins were calculated using optical density and absorbance through UV-VIS spectrophotometer.
Data exclusions	No data was excluded.
Replication	The results of the biochemical experiments could be confirmed by multiple successful replications, as detailed in Methods and the figure legends.
Randomization	No randomization was applied.
Blinding	No blinding was applied.

Reporting for specific materials, systems and methods

We require information from authors about some types of materials, experimental systems and methods used in many studies. Here, indicate whether each material, system or method listed is relevant to your study. If you are not sure if a list item applies to your research, read the appropriate section before selecting a response.

Materials & experimental systems

n/a	Involved in the study	
☐	☒	Antibodies
☒	☐	Eukaryotic cell lines
☒	☐	Palaeontology and archaeology
☒	☐	Animals and other organisms
☒	☐	Clinical data
☒	☐	Dual use research of concern
☒	☐	Plants

Methods

n/a	Involved in the study	
☒	☐	ChIP-seq
☒	☐	Flow cytometry
☒	☐	MRI-based neuroimaging

Antibodies

Antibodies used	Anti-hexahistidine tag lgG antibody (Host: rabbit, Clonality: polyclonal, Application: western Blot, Catalog number: GTX115045, Supplier: GenTex, 10,000 dilution)<br>HRP-conjugated anti-rabbit lgG antibody for His-tag (Host: goat, Clonality: polyclonal, Application: western Blot, Catalog number: GTX213110-01, Supplier: GenTex, 7500 dilution)<br>Anti-trihemagglutinin tag lgG antibody (Host: mouse, Clonality: monoclonal, Application: western Blot, Catalog number: SC-7392, Supplier: Santa Cruze, 10,000 dilution)<br>HRP-conjugated anti-mouse lgG antibody for HA-tag (Host: goat, Clonality: polyclonal, Application: western Blot, Catalog number: SC-2005, Supplier: Santa Cruze, 7500 dilution)
Validation	All the commercial antibodies were verified by the manufacturers according to immunoblots and/or images on their websites.

Plants

Seed stocks	*Report on the source of all seed stocks or other plant material used. If applicable, state the seed stock centre and catalogue number. If plant specimens were collected from the field, describe the collection location, date and sampling procedures.*
Novel plant genotypes	*Describe the methods by which all novel plant genotypes were produced. This includes those generated by transgenic approaches, gene editing, chemical/radiation-based mutagenesis and hybridization. For transgenic lines, describe the transformation method, the number of independent lines analyzed and the generation upon which experiments were performed. For gene-edited lines, describe the editor used, the endogenous sequence targeted for editing, the targeting guide RNA sequence (if applicable) and how the editor was applied.*
Authentication	*Describe any authentication procedures for each seed stock used or novel genotype generated. Describe any experiments used to assess the effect of a mutation and, where applicable, how potential secondary effects (e.g. second site T-DNA insertions, mosiacism, off-target gene editing) were examined.*
